# Does BMI Impact Outcomes in Patients Undergoing Open Abdominal Wall Reconstruction? A Systematic Review and Meta‐Analysis

**DOI:** 10.1002/wjs.12649

**Published:** 2025-06-17

**Authors:** Syed Ali Farhan, Syed Husain Farhan, Jeffrey E. Janis

**Affiliations:** ^1^ Department of General Surgery Harlem Hospital Center New York New York USA; ^2^ Department of Surgery Dow University of Health Sciences Karachi Pakistan; ^3^ Department of Plastic & Reconstructive Surgery College of Medicine The Ohio State University Wexner Medical Center Columbus Ohio USA

**Keywords:** abdominal wall reconstruction, BMI, complex ventral hernia repair, obesity, recurrence

## Abstract

**Importance:**

Obesity is a significant factor that increases complication rates in patients undergoing abdominal wall reconstruction (AWR). This has led to caution about performing elective AWR in patients with higher body mass index (BMI). In light of this, our study aims to synthesize the current information on AWR outcomes in patients stratified according to the obesity classification, providing evidence‐based insights into the impact of BMI on AWR outcomes.

**Objective:**

To compare the clinical outcomes in patients of different BMI groups undergoing AWR.

**Data Sources:**

A systematic literature search of two databases (PubMed and Cochrane CENTRAL) from January 1st, 1966, until July 31, 2024, identified five relevant studies.

**Study Selection:**

Included in our analysis were original studies that assessed clinical outcomes in patients with a BMI < 35 kg/m^2^ compared to those with a BMI ≥ 35 kg/m^2^ undergoing elective AWR. Studies with a patient population of less than 18 years or oncologic patient population were excluded.

**Data Extraction and Synthesis:**

This systematic review and meta‐analysis are reported as per the PRISMA statement. As recommended by the Cochrane Collaboration, the Newcastle–Ottawa scale was used to evaluate methodological quality. The Mantel–Haenszel random‐effects method was used to calculate the pooled odds ratios (ORs) with their 95% confidence intervals (CIs).

**Main Outcome:**

The primary outcomes were hernia recurrence, readmission, reoperation, and surgical site infection (SSI).

**Results:**

Out of 4769 classifiable patients that underwent AWR, the majority were obese‐ CDC Class 1, 2 (2401; 50%) or morbidly obese‐Class 3 (1054; 22%). Patients with a BMI < 35 kg/m^2^ compared to a BMI ≥ 35 kg/m^2^ were associated with significantly decreased odds of readmission (OR 0.52, 95% CI 0.38–0.70, *I* ^2^ = 0%, and *p* < 0.0001), reoperation (OR 0.72, 95% CI 0.55–0.93, *I* ^2^ = 17%, and *p* = 0.01), and developing SSI (OR 0.62, 95% CI 0.48–0.81, *I* ^2^ = 35%, and *p* = 0.0005), whereas hernia recurrence (OR 1.03, 95% CI 0.35–3.00, *I* ^2^ = 88%, and *p* = 0.96) was statistically insignificant.

**Conclusion and Relevance:**

A patient's BMI should not be the sole determinant when planning elective AWR, as increasing BMI does not impact hernia recurrence rates. However, obese patients should be counseled on the higher risk of developing infections, requiring reoperation, and necessitating readmission due to their weight.

## Graphical abstract

This PRISMA flowchart outlines the study selection process for a systematic review and meta‐analysis investigating the impact of BMI on outcomes following open abdominal wall reconstruction. Of the 1,696 records initially identified, 1,196 were screened after duplicate removal. Following full‐text assessment of 181 articles, 5 studies met the eligibility criteria and were included in both the qualitative and quantitative synthesis.

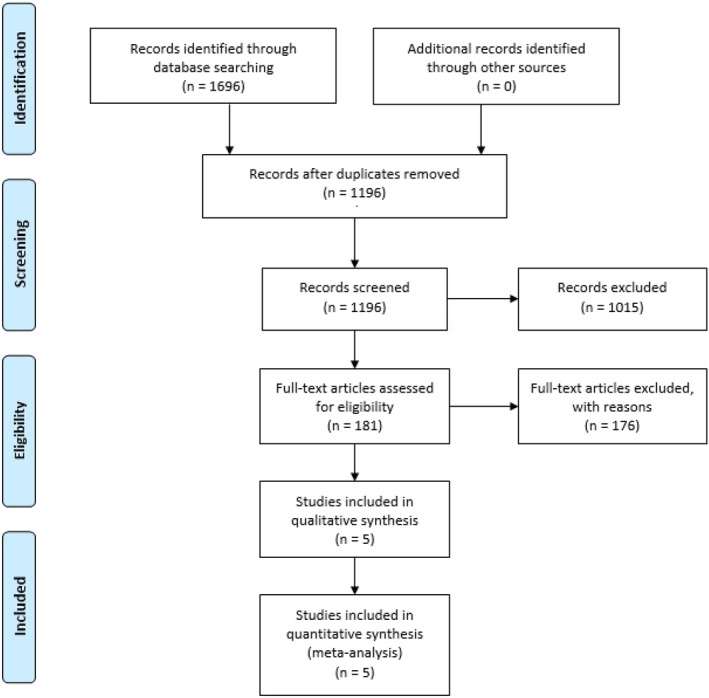

**Summary**

- *Question:* Is BMI an independent risk factor for hernia recurrence and other complications in patients undergoing abdominal wall reconstruction (AWR)? If so, what BMI threshold ensures safe AWR for ventral hernia repair? Additionally, is there variation in complication risks among patients with different classes of obesity?
- *Finding:* Our analysis revealed that patients with a BMI < 35 kg/m^2^ had significantly lower odds of readmission, reoperation, and developing surgical site infections (SSI) compared to those with a BMI ≥ 35 kg/m^2^. However, the difference in hernia recurrence rates was not statistically significant.
- *Meaning:* An increase in BMI does not correspond to a higher risk of hernia recurrence in patients undergoing AWR. However, a higher BMI is associated with a greater likelihood of postoperative complications, such as SSI, and an increased risk of readmissions and reoperations. Therefore, obese patients should be counseled regarding the possible risks before undergoing AWR.

## 1 Introduction

Obesity is an omnipresent challenge faced by the healthcare community in the United States, with its prevalence predicted to reach 40% by 2030 [1]. Obesity, based on the body mass index (BMI), is generally categorized into the following three classes, that is, Class 1: BMI of 30– < 35 kg/m^2^, Class 2: BMI of 35– < 40 kg/m^2^, and Class 3: BMI of 40 kg/m^2^ or higher [2]. Alarmingly, class 3 obesity, also referred to as “severe obesity,” is increasing among the population at a much faster rate than the lower classes of obesity. As with a myriad of other metabolic and functional disorders, obesity leads to structural weakness of the abdominal myofascia, leading to hernia recurrence [3]. This can eventually lead to cycles of additional recurrences over time, impacting quality of life and generating additional costs to the system [4].

Abdominal wall reconstructions (AWR) are increasingly being performed in the United States and worldwide, with an estimated 400,000 incisional hernia repairs per year, as per the Cochrane collaboration [5]. In 2006, the estimated expenditure on AWRs performed in the United States was $3.2 billion, which has undoubtedly increased in today's age of laparoscopic and robotic surgery [6]. These costs are further driven up by subsequent postoperative complications after AWR, such as surgical site infections (SSI), deep space infections, abscesses, or mesh infection, leading to its breakdown [7]. This can lead to a domino effect whereby more minor complications, such as superficial SSI, can progress to even more significant complications, such as mesh infection requiring mesh removal and subsequent increased risk of recurrence. Holihan et al. called this the “vicious cycle” of hernia repair, leading to complications, reoperation, and rerepair [8].

Obesity has been a major cofactor in increasing complication rates in patients undergoing AWR [9]. As a result, there has been caution, if not reluctance, to perform elective AWR in patients with higher BMI's [10, 11]. Multiple studies have addressed the impact of obesity in patients undergoing AWR [3, 9, 10, 12, 13, 14, 15]. However, the published literature lacks a consensus. In practice, this ambiguity and potential delay in operative management could increase the risk of hernia complications, such as incarceration and strangulation, during the observation period while awaiting weight loss. Therefore, we systematically reviewed studies on performing AWR in obese patients and examined outcomes, such as recurrence, readmission, and reoperation rates, stratified according to the CDC obesity classification. We also performed a meta‐analysis of operative outcomes and complications in obese patients undergoing AWR.

## 2 Methods

### 2.1 Data Sources and Search Strategy

This systematic review and meta‐analysis followed the Preferred Reporting Items for Systematic Reviews and Meta‐Analyses (PRISMA) guidelines [16] and its protocol was registered in PROSPERO (CRD42024533859). A thorough search of Cochrane CENTRAL and PubMed/MEDLINE databases was conducted from January 1st, 1966, until July 31st, 2024, with no restrictions to the publication status. Furthermore, bibliographies of included articles were also screened. Only articles in the English language were eligible for inclusion. The search string was created using PubMed MeSH and related text terms. Keywords used in the search string were “abdominal wall reconstruction,” “body mass index,” and “obesity.” The complete search strategy is presented in Supporting Information S1: Table S1.

### 2.2 Study Selection and Eligibility Criteria

All articles identified from the systematic search were transferred to EndNote version X8.1 (Clarivate Analytics), where duplicates were removed. By utilizing covidence [17], two authors (S.A.F. and S.H.F.) worked collaboratively, independently screening and selecting studies, with any discrepancies being resolved by a third author (J.E.J.). Mendeley reference management software (Mendeley Desktop v 1.19.4, London, UK) facilitated citation management. AWR is defined in our study as ventral hernia repair (VHR) with myofascial release as defined by the Abdominal Core Health Quality Collaborative. This myofascial release includes techniques, such as external oblique myofascial release, otherwise termed the “anterior component separation (ACS)” as well as the posterior rectus sheath myofascial release, otherwise known as the “posterior component separation (PCS)” or transversus abdominis release (TAR) [18]. Those studies assessing outcomes using laparoscopic or robotic techniques were excluded to limit the potential bias caused by different surgical techniques.

All original studies, whether retrospective or prospective, evaluating the impact of different BMI groups on clinical outcomes in patients undergoing open AWR were included. We only included studies that stratified outcomes by BMI classes. Since most studies provided outcome data comparing patients with a BMI ≥ 35 kg/m^2^ to those with a BMI < 35 kg/m^2^, a meta‐analysis was performed for these two groups. Additionally, other BMI groups were also compared systematically to assess clinical outcomes across various BMI categories. If two studies used the same database with overlapping time periods, the study with the greater duration and larger sample size was selected unless it presented new data in the prespecified subgroup analyses. Furthermore, there was much heterogeneity in the reported outcomes, and although “overall,” “major,” “minor,” “wound,” and “infection” complications were reported in some studies, they were not defined as to what constituted these complications. Therefore, we could not include them in our meta‐analysis. Studies with a patient population of less than 18 years of age or assessed outcomes in oncologic patients were excluded. Notably, one study present in the literature had reported their outcomes based on all BMI classes. However, this study could not be included in our systematic review due to the inclusion of oncologic patients in their study population [3]. Moreover, reviews, conference abstracts, editorials, case reports, case series, and those involving animal subjects were excluded from the analysis. Studies in languages other than English were excluded from the final list of included studies.

#### 2.2.1 Data Extraction

Two authors (S.A.F. and S.H.F.) independently evaluated the data and supplementary materials, resolving conflicts through consultation with a third author (J.E.J.). Data extracted from the included studies included the year of publication, number of participants, baseline and operative characteristics of patients, follow‐up duration, and clinical outcomes related to efficacy and safety. The clinical outcomes assessed were (1) hernia recurrence, (2) readmission, (3) reoperation, and (4) surgical site infection (SSI).

#### 2.2.2 Quality Assessment

Two authors (S.H.F. and S.A.F.) performed the quality assessment following the Meta‐Analysis of Observational Studies in Epidemiology (MOOSE) group guidelines. As recommended by the Cochrane Collaboration, the Newcastle–Ottawa scale (NOS) was used to evaluate methodological quality or risk of bias in nonrandomized studies [19, 20]. In cases of disagreement between the two authors, a third author (J.E.J.) was consulted to resolve the issues. The risk assessment rating of each study is presented in Supporting Information S1: Table S2.

### 2.3 Statistical Analysis

We conducted the statistical analysis using Review Manager (RevMan version 5.3; Copenhagen: The Nordic Cochrane Centre, The Cochrane Collaboration, 2014). Results from all studies were combined using the Mantel–Haenszel random‐effects model with a two‐tailed *p* value of less than 0.05, which was considered statistically significant. Odds ratios (ORs) with 95% confidence intervals (CIs) were used for dichotomous outcomes. For a visual representation of the analysis, forest plots were created. Statistical heterogeneity was assessed with the *I* ^2^ index, categorized as low (< 50%), moderate (50%–75%), and high (> 75%) heterogeneity [16].

## 3 Results

### 3.1 Study Characteristics and Baseline Demographics

**FIGURE 1:**
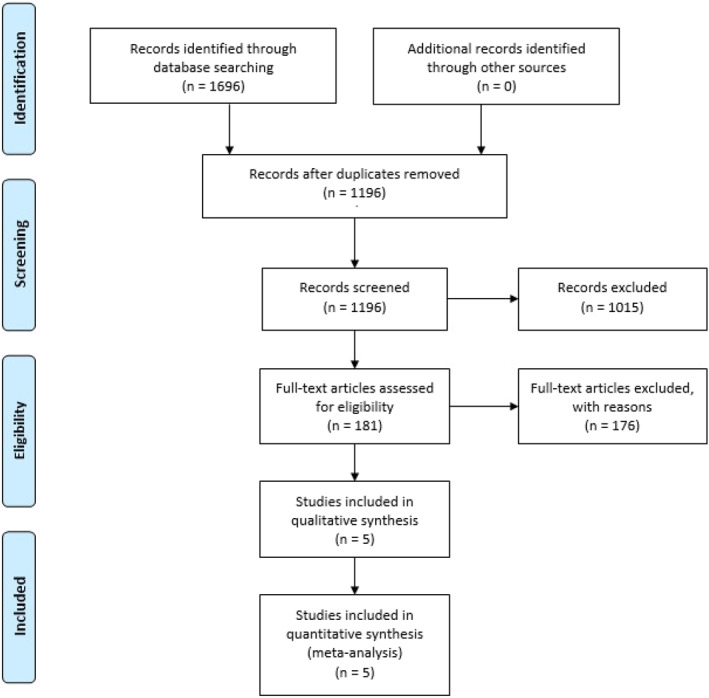
PRISMA flowchart of included studies.

A preliminary search of electronic databases produced 1696 possible articles, of which 1196 were assessed after removing the duplicates. After the abstract screening, 181 articles were checked for eligibility. Finally, five original articles met the eligibility criteria and were included in the qualitative (systematic review) and quantitative analysis, that is, meta‐analysis (Figure 1). All five studies in our analysis reported a low risk of bias on the Newcastle–Ottawa scale (Supporting Information S1: Table S2). The baseline characteristics of the included studies are presented in Table 1.

**TABLE 1:** Overview of study characteristics and baseline demographics.

Study	Country	Design	Mean follow‐up (months)	No. of participants		Mesh type	Position of mesh placement	Type of component separation	Age (avg)		Men, *n* (%)		Women, *n* (%)		Diabetes mellitus, *n* (%)		Smoking *n* (%)		COPD, *n* (%)		Length of stay (days)		ASA class III or IV, *n* (%)		Wound class, *n* (%)	
				Intervention	Control				Intervention	Control	Intervention	Control	Intervention	Control	Intervention	Control	Intervention	Control	Intervention	Control	Intervention	Control	Intervention	Control	Intervention	Control
Maskal et al. 2024 [10]	USA	Prospective cohort	1 month (hernia recurrence follow‐up to 12 months)	340	749	Synthetic mesh	Sublay‐retromuscular	Posterior component separation	58	61	148 (44)	371 (50)	192 (56)	378 (50)	100 (29)	144 (19)	15 (4)	40 (5)	40 (12)	55 (7)	6	5	284 (84)	595 (79)	Clean 1082 (74), clean‐contaminated 220 (15), contaminated 67 (5), and infected 87 (6)	Clean 2352 (78), clean‐contaminated 456 (15), contaminated 103 (3), and infected 104 (3)
Docimo et al. 2019 [13]	USA	Retrospective cohort	1 month	1456	3015	N/A	N/A	Open component separation (nonspecified)	N/A	N/A	520 (35)	1626 (54)	936 (65)	1389 (46)	410 (28)	433 (14)	280 (19)	699 (23)	102 (7)	162 (5)	N/A	N/A	962 (66)	1442 (47)	N/A	N/A
Smolevitz et al. 2018 [15]	USA	Retrospective cohort	14 months	60	125	Bioprosthetic and synthetic	Underlay	Anterior component separation	52	57	14 (22)	59 (47)	46 (78)	66 (53)	N/A	N/A	N/A	N/A	N/A	N/A	11.2	10.6	N/A	N/A	N/A	N/A
Desai et al. 2016 [12]	USA	Prospective cohort	16 months	89	224	Bioprosthetic and synthetic	N/A	Component separation (nonspecified)	53	53	26 (29)	97 (43)	63 (71)	127 (56)	29 (32)	49 (21)	21 (24)	44 (20)	N/A	N/A	N/A	N/A	N/A	N/A	N/A	N/A
Nelson et al. 2014 [21]	USA	Retrospective cohort	1 month	314	614	Bioprosthetic and synthetic	N/A	Component separation (nonspecified)	53	57	228 (73)	273 (44)	86 (27)	341 (56)	220 (70)	547 (89)	57 (18)	173 (28)	16 (5)	33 (5)	9.4	7.3	245 (78)	279 (45)	Clean 211 (67), clean‐contaminated 59 (19), contaminated 18 (6), and dirty 26 (8)	Clean 414 (67), clean‐contaminated 116 (19), contaminated 46 (7), and dirty 38 (6)

*Note:* The intervention arm was a BMI ≥ 35 kg/m2, and the control arm was a BMI < 35 kg/m2.

### 3.2 Delineation of Outcomes According to Respective Studies

To determine which outcomes to include in our meta‐analysis, we outlined the final studies included in our systematic review. We categorized all the studies based on the reported outcomes and their follow‐up periods as shown in Table 2. Two studies reported outcomes at 30 days [21], one study at 90 days [10], and two studies did not specify the time point at which they reported their respective outcomes [12, 15].

**TABLE 2:** Delineation of studies by outcomes.

Study	Outcomes of interest	Number of postop days at which outcomes/Complications reported
Docimo et al. 2019 [13]	SSI, reoperation, readmission, minor complication, and major complication	30 days
Maskal et al. 2024 [10]	Recurrence (stratified according to the number of years postop), SSO, SSI, SSOPI, readmission, reoperation, mesh infection, and LOS	90 days
Smolevitz et al. 2018 [15]	Recurrence (not stratified according to the number of years postop), wound complications, postoperative complication, LOS, and SSI	Not available
Nelson et al. 2014 [21]	Wound infection, reoperation, major surgical complication, major medical complication, and major renal complications	30 days
Desai et al. 2016 [12]	Overall complications, recurrence, reoperations, and infection	Not available

### 3.3 Meta‐Analysis of Clinical Outcomes

**FIGURE 2:**
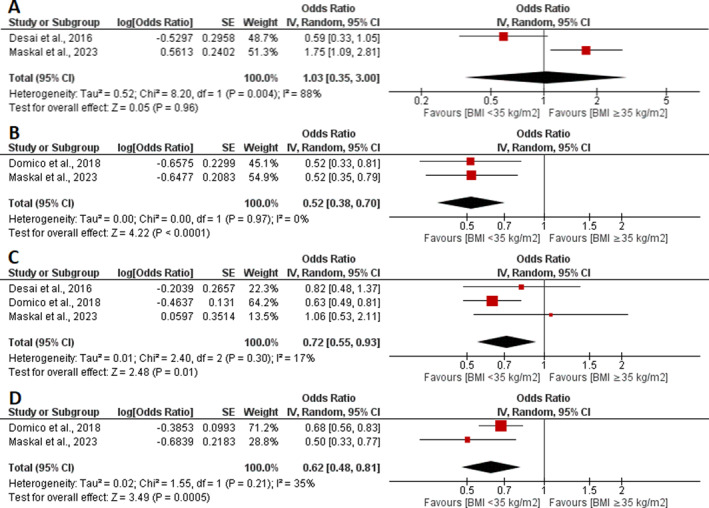
Forest plot of (A) hernia recurrence, (B) readmission, (C) reoperation, and (D) surgical site infection stratified by a BMI < 35 kg/m^2^ and a BMI ≥ 35 kg/m^2^.

We systematically reviewed each outcome to see how it was reported in each included study and evaluated it based on BMI classes. Of the five included studies, only one study [10] stratified their reported outcomes based on all BMI classes. There was much heterogeneity in reporting outcomes by respective studies regarding the BMI classification. Therefore, to systematically delineate our outcomes of interest, we stratified these into three main categories: (1) BMI < 35 kg/m^2^ and BMI ≥ 35 kg/m^2^, (2) BMI < 40 kg/m^2^ and BMI ≥ 40 kg/m^2^, and (3) BMI < 30 kg/m^2^ and BMI ≥ 40 kg/m^2^ as shown in Table 3. For the meta‐analysis, the BMI < 35 kg/m2 and BMI ≥ 35 kg/m2 groups were only analyzed, as the other two categories were reported by only one study each. Therefore, a systematic analysis was performed for a BMI < 40 kg/m^2^ and a BMI ≥ 40 kg/m^2^ and a BMI < 30 kg/m^2^ and a BMI ≥ 40 kg/m^2^ categories as shown in Table 3. The meta‐analysis is shown in Figure 2A–D and described below in detail.

**TABLE 3:** Quantitative evaluation of clinical outcomes of individual studies based on the reported classes of BMI; (A) surgical site infection, (B) reoperation, (C) readmission, and (D) hernia recurrence.

Study	S. No	(A) Surgical site infection					
	1.	BMI < 35 kg/m^2^			BMI ≥ 35 kg/m^2^		
		Events	Total	Percentage	Events	Total	Percentage
Desai et al. 2016 [12]		N/A					
Docimo et al. 2019 [13]^a^		N/A					
Maskal et al. 2024 [10]		51	749	6.80%	43	340	12.64%

	2.	BMI < 40 kg/m^2^			BMI ≥ 40 kg/m^2^		
		Events	Total	Percentage	Events	Total	Percentage
Smolevitz et al. 2018 [15]		N/A					

	3.	BMI < 30 kg/m^2^			BMI ≥ 40 kg/m^2^		
		Events	Total	Percentage	Events	Total	Percentage
Nelson et al. 2014 [21]		41	614	6.67%	41	314	13.05%

Study	S. No	(B) Reoperation					
	1.	BMI < 35 kg/m^2^			BMI ≥ 35 kg/m^2^		
		Events	Total	Percentage	Events	Total	Percentage
Desai et al. 2016 [12]		68	224	30.35%	31	89	34.83%
Docimo et al. 2019 [13]^a^		N/A					
Maskal et al. 2024 [10]		28	749	3.73%	12	340	3.52%

	2.	BMI < 40 kg/m^2^			BMI ≥ 40 kg/m^2^		
		Events	Total	Percentage	Events	Total	Percentage
Smolevitz et al. 2018 [15]		N/A					

	3.	BMI < 30 kg/m^2^			BMI ≥ 40 kg/m^2^		
		Events	Total	Percentage	Events	Total	Percentage
Nelson et al. 2014 [21]		33	614	5.37%	38	314	12.10%

Study	S. No	(C) Readmission					
	1.	BMI < 35 kg/m^2^			BMI ≥ 35 kg/m^2^		
		Events	Total	Percentage	Events	Total	Percentage
Desai et al. 2016 [12]		N/A					
Docimo et al. 2019 [13]^a^		N/A					
Maskal et al. 2024 [10]		58	749	7.74%	47	340	13.82%

	2.	BMI < 40 kg/m^2^			BMI ≥ 40 kg/m^2^		
		Events	Total	Percentage	Events	Total	Percentage
Smolevitz et al. 2018 [15]		N/A					

	3.	BMI < 30 kg/m^2^			BMI ≥ 40 kg/m^2^		
		Events	Total	Percentage	Events	Total	Percentage
Nelson et al. 2014 [21]		N/A					

Study	S. No	(D) Hernia recurrence					
	1.	BMI < 35 kg/m^2^			BMI ≥ 35 kg/m^2^		
		Events	Total	Percentage	Events	Total	Percentage
Desai et al. 2016 [12]		40	224	17.85%	24	89	26.96%
Docimo et al. 2019 [13]^a^		N/A					
Maskal et al. 2024 [10]		88	749	11.74%	24	340	7.05%

	2.	BMI < 40 kg/m^2^			BMI ≥ 40 kg/m^2^		
		Events	Total	Percentage	Events	Total	Percentage
Smolevitz et al. 2018 [15]		3	125	2.40%	4	60	6.66%

	3.	BMI < 30 kg/m^2^			BMI ≥ 40 kg/m^2^		
		Events	Total	Percentage	Events	Total	Percentage
Nelson et al. 2014 [21]		N/A					

^a^ Docimo et al. provided data in the form of odds ratios (ORs) for all clinical outcomes.

#### 3.3.1 Hernia Recurrence

Two studies [10, 13] assessed the hernia recurrence outcome with 973 patients in the BMI < 35 kg/m^2^ group and 429 patients in the BMI ≥ 35 kg/m^2^ group. The cumulative estimate was statistically insignificant between the two groups (OR 1.03, 95% CI 0.35–3.00, and *p* = 0.96) (Figure 2A). However, this finding was associated with high heterogeneity (*I* ^2^ = 88%), and because only two studies reported the outcome, it was not possible to conduct a sensitivity analysis (Figure 2A).

#### 3.3.2 Readmission

Two studies reported this outcome [10, 13] with 3764 patients in the BMI < 35 kg/m^2^ group and 1796 patients in the BMI ≥ 35 kg/m^2^ group. There was a statistically significant decrease in readmission in patients in the BMI < 35 kg/m^2^ group as compared to patients in the BMI ≥ 35 kg/m^2^ group (OR 0.52, 95% CI 0.38–0.70, and *p* < 0.0001; Figure 2B). The heterogeneity in this outcome measured was low (*I* ^2^ = 0%; Figure 2B).

#### 3.3.3 Reoperation

A total of three studies [10, 12, 13] measured this outcome with 3988 patients in the BMI < 35 kg/m^2^ group and 1885 patients in the BMI ≥ 35 kg/m^2^ group. A statistically significant decrease in reoperations was observed in the BMI < 35 kg/m^2^ group as compared to the BMI ≥ 35 kg/m^2^ group (OR 0.72, 95% CI 0.55–0.93, and *p* = 0.01; Figure 2C). The value of heterogeneity was low in this outcome (*I* ^2^ = 17%; Figure 2C).

#### 3.3.4 Surgical Site Infection

Two studies [10, 13] assessed this outcome with 3764 patients in the BMI < 35 kg/m^2^ group and 1796 patients in the BMI ≥ 35 kg/m^2^ group. There was a statistically significant decrease in surgical site infections in patients in the BMI < 35 kg/m^2^ group as compared to patients in the BMI ≥ 35 kg/m^2^ group (OR 0.62, 95% CI 0.48–0.81, and *p* = 0.0005; Figure 2D). The heterogeneity observed in this outcome was low (*I* ^2^ = 35%; Figure 2D).

## 4 Discussion

This is the first review paper that systematically delineates and analyzes whether obesity, stratified by CDC class, affects outcomes in AWR. Intriguingly, and refuting our original hypothesis, the primary finding of our meta‐analysis shows that a BMI ≥ 35 kg/m^2^ does not increase the chances of developing hernia recurrence in patients undergoing AWR. Even though the heterogeneity of this result is high (*I* ^2^ = 88%), it is interesting to see the two studies [10, 12] pooled together gave contrasting results for recurrence rates in patients with a BMI > 35 kg/m^2^. In our systematic review, Desai et al. [12] reported recurrence rates of 27% for patients with a BMI > 35 kg/m^2^, whereas Maskal et al. [10] reported a recurrence of 12%. The diversity in surgical technique, mesh usage, and type may be behind the cause of this discrepancy as pointed out by Maskal et al., who used synthetic mesh and performed TAR in all their patients. More than a quarter (28%) of the patients did not receive mesh placement and half did not undergo component separation in AWR as described by Desai et al. However, another caveat to the discrepancy in recurrence rates between the two studies is the difference in duration of follow‐up, that is, Maskal et al.—3 months versus Desai et al.—> 12 months. Hernia recurrence is a time‐dependent complication and may not manifest in studies with shorter time durations of patient follow‐up. Singhal et al. reported an average of 16 months to first recurrence after the initial repair [22] whereas, Bhardwaj et al. reporting, on a larger subset of patients, reported increasing ventral hernia recurrence rates up to 5 years status‐post the index repair [4]. Therefore, long‐term follow‐up of preferably more than a year is essential to monitor for recurrence and other complications in obese patients undergoing AWR.

Our study aims to clarify the morbidity associated with performing elective AWR in obese patients. The pooled results of our meta‐analysis demonstrate that readmissions and reoperations increased in patients with a BMI of ≥ 35 kg/m^2^. The heterogeneity of the pooled data regarding readmission and reoperation was low, indicating the strength of the analysis. The most common causes for readmissions and subsequent readmissions in AWR are wound‐related complications such as wound dehiscence and SSIs [10]. Indeed, our meta‐analysis also showed that SSIs increase in patients with a BMI of ≥ 35 kg/m^2^ undergoing AWR after pooling two studies that unanimously reported this in each of their respective papers [10, 13]. Giordano et al. [3] also reported higher rates of seroma, wound dehiscence, infection, and fat necrosis in patients with a BMI ≥ 35 kg/m^2^.

Component separation in obese patients may well be required on a more frequent basis due to the nature of the operative anatomy of the patient, as it is often challenging to achieve primary fascial closure in patients with high intra‐abdominal pressures due to excessive visceral fat [14]. Quintessentially, all the studies included in our review employed component separation while performing AWR; however, only two studies explicitly mentioned the type of CS (one anterior CS [15] and one posterior CS/TAR [10]). CS leads to reduced hernia recurrence; however, it can predispose to increased wound complications if wide‐undermining is performed rather than perforator‐sparing minimally invasive approaches [23]. Posterior CS, also known as TAR, has been reported to be superior to anterior CS regarding operative outcomes and complications [23, 24]. However, the success of the perforator‐sparing anterior CS technique in optimized patients should not be underestimated, as it equalizes complication rates between the two approaches [25, 26]. Comparison of how different CS techniques impact outcomes in patients with a BMI > 30 kg/m^2^ is beyond the scope of this study and may be a topic of future studies.

Obesity, as a standalone clinical factor, should not be a contraindication to perform or delay ventral hernia repair to achieve weight loss before surgery. Delaying ventral hernia repair or “watchful waiting” may increase the propensity of developing complications such as fistulas, perforation, or even incarceration [27, 28]. Individual surgeons may adopt a cutoff value above which they do not perform AWR or complex hernia repair based on personal preferences and experiences. Furthermore, expert consensus on ventral hernia does not recommend performing repairs in patients with a BMI > 50 kg/m^2^ [11]. Therefore, patients with BMIs between 30 kg/m^2^ and 50 kg/m^2^ must be optimized for the procedure on a case‐by‐case basis, based on the risk of developing complications versus the benefits of achieving weight loss before surgery.

Medical optimization (or prehabilitation) is the first line; however, it may take time. Rosen et al. published promising weight loss results when they collaborated with a weight loss specialist and adopted a protein‐sparing diet for their patients over 3 months [29]. Cattaneo et al. also achieved satisfactory “optimization” of their patients to undergo AWR by having them participate in supervised exercise programs, nutritional and smoking cessation counseling, and psychological support [30]. Performing bariatric surgery before hernia repair reduces hernia recurrence rates and can be discussed with the patient if timely medical weight loss is not realistic [31, 32]. Concurrent panniculectomy with AWR is also a surgical option that can be utilized selectively. Intraoperatively, this may enhance exposure and reduce strain on the fascia postoperatively, leading to reduced recurrence. However, the risk of wound complications and SSI should be considered when planning this surgical approach [31, 33]. The use of closed‐incision negative pressure therapy has also been shown to mitigate wound complications in these scenarios [33].

The effect of body fat distribution may have been understudied and needs more consideration when considering ventral hernia development and its subsequent repair. Specifically, studies have shown an increased risk of ventral hernia development in patients with increased content of visceral fat distribution [34, 35, 36]. BMI, although the most common indicator of obesity, is a poor indicator of body fat distribution and has started to fall out of favor in the broader medical community [37]. Recent studies have shown that the waist‐to‐hip ratio is a more predictive metric of complications after various surgeries [34, 38, 39, 40]. Another useful adjunct could be perioperative computed tomography (CT) scanning in patients with high BMI to assess their body fat distribution. Winters et al. obtained different body fat metrics utilizing CT scans in complex ventral hernia repair patients to predict hernia recurrence and SSI [41]. In AWR, studies have shown that CT scans can aid perioperative planning based on deep learning data models that can analyze imaging to predict outcomes [42, 43].

Some limitations should be considered when interpreting our findings. All the studies included were observational, which has a greater risk of bias and confounding compared to RCTs. Due to a limited number of studies, sensitivity analyses, subgroup analyses, and publication bias assessments were not possible. Only two studies were pooled with short follow‐up periods reporting on hernia recurrence. This may introduce a follow‐up bias in our findings due to insufficient time to observe long‐term hernia recurrence risk in the participants. This study‐level meta‐analysis did not account for significant variations across studies, indicating a need for patient‐level meta‐analysis.

## 5 Conclusion

Our study shows that obesity, in grades of increasing BMI, does not translate into an increased risk of hernia recurrence in patients undergoing AWR. However, BMI does lead to an increased risk of short‐term complications, such as infections, and therefore, obese patients should be counseled that there is an increased risk of readmissions and even reoperations. Considering the current data, we recommend that no singular BMI cutoff be used to deny patients AWR and that a holistic approach be adopted by surgeons in counseling patients regarding the risks and benefits of undergoing AWR. Furthermore, instead of solely relying on BMI, additional metrics, such as waist‐to‐hip ratio and CT imaging, should be employed in obese patients for better perioperative planning. In patients deemed truly unfit for the procedure due to their multiple comorbidities, medical and surgical optimization of their weight can be undertaken to make them a suitable surgical candidate for AWR. Large prospective studies that enroll patients of all BMI classes to compare clinical outcomes and complications in patients undergoing AWR would be beneficial. A review study that discusses the benefits and efficacy of weight loss strategies before hernia repair or AWR would also be valuable for surgical education.

## Author Contributions

**Syed Ali Farhan:** conceptualization, data curation, investigation, methodology, project administration, supervision, visualization, writing – original draft, writing – review and editing. **Syed Husain Farhan:** conceptualization, data curation, formal analysis, methodology, software, writing – original draft, writing – reviewing and editing. **Jeffrey E. Janis:** conceptualization, methodology, project administration, supervision, validation, writing – review and editing.

## Conflicts of Interest

Dr. Janis receives royalties from Thieme and Springer Publishing. The remaining authors declare no conflicts of interest.

## Supporting information

Supporting Information S1
