# Supplementary material for: Does BMI Impact Outcomes in Patients Undergoing Open Abdominal Wall Reconstruction? A Systematic Review and Meta‐Analysis
Source: World J Surg. 2025 Jun 17;49(7):1777–86. doi: 10.1002/wjs.12649 (PMC12282558; doi:10.1002/wjs.12649)
Supplement: Supplementary file 1 — Supporting Information S1 [file WJS-49-1777-s001.docx]

**Does BMI Impact Outcomes in Patients Undergoing Open Abdominal Wall Reconstruction? A Systematic Review and Meta-analysis.**

Authors: Syed Ali Farhan^1^, MBBS; Syed Husain Farhan^2^, MBBS; Jeffrey E. Janis^3^, MD, FACS

Affiliations:

1. Department of General Surgery, Harlem Hospital Center, New York, NY, 10027
2. Department of Surgery, Dow University of Health Sciences, Karachi, Pakistan
3. Department of Plastic and Reconstructive Surgery, Ohio State University Wexner Medical Center, Columbus, OH, 43210

| **Supplementary Table 1: Delineation of Searched Databases and Search Strategies** | | |
| --- | --- | --- |
| DATABASE | SEARCH STRATEGY | ARTICLES |
| PUBMED | ("abdominoplasty"[MeSH Terms] OR "abdominoplasty"[All Fields] OR ("abdominal"[All Fields] AND "wall"[All Fields] AND "reconstruction"[All Fields]) OR "abdominal wall reconstruction"[All Fields] OR (("complex"[All Fields] OR "complex s"[All Fields] OR "complexant"[All Fields] OR "complexants"[All Fields] OR "complexated"[All Fields] OR "complexation"[All Fields] OR "complexations"[All Fields] OR "complexe"[All Fields] OR "complexed"[All Fields] OR "complexes"[All Fields] OR "complexing"[All Fields] OR "complexities"[All Fields] OR "complexity"[All Fields] OR "complexs"[All Fields]) AND ("abdominoplasty"[MeSH Terms] OR "abdominoplasty"[All Fields] OR ("abdominal"[All Fields] AND "wall"[All Fields] AND "reconstruction"[All Fields]) OR "abdominal wall reconstruction"[All Fields])) OR (("complex"[All Fields] OR "complex s"[All Fields] OR "complexant"[All Fields] OR "complexants"[All Fields] OR "complexated"[All Fields] OR "complexation"[All Fields] OR "complexations"[All Fields] OR "complexe"[All Fields] OR "complexed"[All Fields] OR "complexes"[All Fields] OR "complexing"[All Fields] OR "complexities"[All Fields] OR "complexity"[All Fields] OR "complexs"[All Fields]) AND ("hernia, ventral"[MeSH Terms] OR ("hernia"[All Fields] AND "ventral"[All Fields]) OR "ventral hernia"[All Fields] OR ("ventral"[All Fields] AND "hernia"[All Fields])) AND ("repairability"[All Fields] OR "repairable"[All Fields] OR "repaire"[All Fields] OR "repaired"[All Fields] OR "repairment"[All Fields] OR "wound healing"[MeSH Terms] OR ("wound"[All Fields] AND "healing"[All Fields]) OR "wound healing"[All Fields] OR "repair"[All Fields] OR "repairing"[All Fields] OR "repairs"[All Fields]))) AND ("BMI"[All Fields] OR ("obeses"[All Fields] OR "obesity"[MeSH Terms] OR "obesity"[All Fields] OR "obese"[All Fields] OR "obesities"[All Fields] OR "obesity s"[All Fields]) OR ("obeses"[All Fields] OR "obesity"[MeSH Terms] OR "obesity"[All Fields] OR "obese"[All Fields] OR "obesities"[All Fields] OR "obesity s"[All Fields]) OR ("body mass index"[MeSH Terms] OR ("body"[All Fields] AND "mass"[All Fields] AND "index"[All Fields]) OR "body mass index"[All Fields])) | 1151 |
| COCHRANE CENTRAL | (abdominal wall reconstruction OR complex abdominal wall reconstruction OR complex ventral hernia repair) AND (BMI OR obesity OR obese OR body mass index) | 545 |

| **Supplementary Table 2: Newcastle- Ottawa Risk Assessment of Included Articles** | | | | | |
| --- | --- | --- | --- | --- | --- |
| NOS Items | Maskal et al., 2023 | Domico et al., 2018 | Smolevitz et al., 2017 | Desai et al., 2016 | Nelson et al., 2014 |
| Representativeness of the exposed cohort (maximum score =1) | 1 | 1 | 1 | 1 | 1 |
| Selection of the non-exposed cohort (maximum score =1) | 1 | 1 | 1 | 1 | 1 |
| Ascertainment of exposure (maximum score =1) | 1 | 1 | 1 | 1 | 1 |
| Demonstration that outcome of interest was not present at start of study (maximum score =1) | 1 | 1 | 1 | 1 | 1 |
| Comparability of cohorts on the basis of the design or analysis controlled for confounders (maximum score =2) | 2 | 2 | 0 | 1 | 2 |
| Assessment of outcome (maximum score =1) | 1 | 1 | 1 | 0 | 1 |
| Was follow-up long enough for outcomes to occur (maximum score =1) | 1 | 1 | 1 | 1 | 1 |
| Adequacy of follow-up of cohorts (maximum score =1) | 1 | 1 | 1 | 1 | 1 |
| **Total Score** | **9** | **9** | **7** | **7** | **9** |

Supplementary Table 1: Overview of Searched Databases and Search Strategi

Supplementary Table 2: Newcastle-Ottawa Risk Assessment for Included Articles
